# Retraction: HSP70 Domain II of *Mycobacterium tuberculosis* Modulates Immune Response and Protective Potential of F1 and LcrV Antigens of *Yersinia pestis* in a Mouse Model

**DOI:** 10.1371/journal.pntd.0012406

**Published:** 2024-08-09

**Authors:** The PLOS Neglected Tropical Diseases Editors

After this article [1] was published concerns were raised regarding Figs 4, 5 and 8.

Specifically:

- In Fig 8, the following panels appear to overlap or partially overlap:
  ○ In Fig 8a, panels B, C and D
  ○ In Fig 8a, the left side of panel L and right side of panel M
  ○ In Fig 8b, panels C and G, and panel F with altered aspect ratio
  ○ In Fig 8c, panels A and K
  ○ In Fig 8c, panels D, E and F
  ○ In Fig 8c, panel J, and in Fig 8d, panel A
  ○ In Fig 8c, panel M, and in Fig 8d, panel K with altered aspect ratio
  ○ In Fig 8d, panels E and F with altered exposure
  ○ In Fig 8d, panels J, L and M
- In Figs 4 and 5 the gating strategy for the analysis of the FACS data is not uniform for all samples as would be expected when directly comparing different experimental conditions.

The corresponding author stated that the original images underlying Fig 8 are unavailable. In the absence of these data, the issues with Fig 8 cannot be resolved. The corresponding author provided the underlying data for the flow cytometry dot plot panels shown in Figs 4 and 5 and the quantitative data underlying the associated bar charts. Regarding Figs 4 and 5, the corresponding author confirmed that the gating strategies were not uniform. They stated this was because the flow cytometry work was performed at different times, and also stated that the gating was set according to the control for each flow cytometry experiment. No additional control flow cytometry data were provided.

In light of the unresolved concerns that question the reliability and validity of the reported results and conclusions, the *PLOS Neglected Tropical Diseases Editors* retract this article.

PLOS noted that the survival study in [1] uses death as an experimental endpoint. The corresponding author provided the following additional information: Mice were monitored daily for weight loss and twice daily for behavior monitoring (clinical score) for up to 14 days. The following humane endpoint criteria were applied: >25% weight loss, blackening of the skin, difficulty in the movement. Mice with a clinical score of 3 were euthanized by CO_2_ (3–4 liters per minute) followed by a secondary method such as cervical dislocation.

LB and SKV did not agree with the retraction and stand by the article’s findings. DPN, NS, PP, SCP, and UT either did not respond directly or could not be reached.
